# Seeking Maxwell’s Demon in a non-reciprocal quantum ring

**DOI:** 10.1038/s41598-019-45583-4

**Published:** 2019-06-25

**Authors:** Aram Manaselyan, Wenchen Luo, Daniel Braak, Tapash Chakraborty

**Affiliations:** 1 Department of Solid State Physics, Yerevan State University, 0025 Yerevan, Armenia; 2 School of Physics and Electronics, Central South University, Changsha, Hunan 410083 P.R. China; 3 Max-Planck Institut für Festkörperforschung, Heisenbergstraße 1, 70569 Stuttgart, Germany; 4 Department of Physics and Astronomy, University of Manitoba, Winnipeg, R3T 2N2 Canada

**Keywords:** Electronic properties and materials, Surfaces, interfaces and thin films

## Abstract

A non-reciprocal quantum ring, where one arm of the ring contains the Rashba spin-orbit interaction but not in the other arm, is found to posses very unique electronic properties. In this ring the Aharonov-Bohm oscillations are totally absent. That is because in a magnetic field the electron stays in the non-Rashba arm, while it resides in the Rashba arm for zero (or negative) magnetic field. The average kinetic energy in the two arms of the ring are found to be very different. It also reveals different “spin temperature” in the two arms of the non-reciprocal ring. The electrons are sorted according to their spins in different regions of the ring by switching on and off (or reverse) the magnetic field, thereby creating order without doing work on the system. This resembles the action of a demon in the spirit of Maxwell’s original proposal, exploiting a non-classical internal degree of freedom. Our demon clearly demonstrates some of the required features on the nanoscale.

## Introduction

In the middle of nineteenth century, physicists were grappling with the implications of the newly established second law of thermodynamics, whose one profound proclamation was that, it is not possible for heat to spontaneously flow from a cold system to a hot system without external work being done on the system (Clausius statement). Then in 1867, James Clerk Maxwell questioned^1^ if the above statement was true only for a system whose properties were governed by the average behavior of the particles or whether it is still valid even at the level of individual particles. In order to test this idea, Maxwell conceived the famous *gedankenexperiment* where a tiny intelligent being (*demon*) with exceptional capacity of being able to observe individual molecules and their speed, was assigned a very special job. The demon was to sort, in a closed box of uniformly distributed particles, the fast (hot) particles and slow (cold) particles in two compartments of the box separated by a wall with a tiny trap door. As time passes, the demon opens or shuts the door to allow the fast or slow particles in their respective compartments, thereby creating a temperature gradient from the initial uniform temperature without doing work on the system and thus violate the second law of thermodynamics^2,3^.

Given the paucity of intelligent demons, for 150 years Maxwell’s demon remained an interesting idea that could never be implemented in a real world. However, in recent years, rapid progress in nanoscale physics has resulted in devices where the dynamics of even individual electron can be controlled. Examples of such systems are quantum dots (artificial atoms)^4–9^ and quantum rings^10–13^. Quite naturally, this demon with incredible dexterity has now made a remarkable comeback in nanoscale devices. Theoretical studies indicate that the demon is lurking in quantum dots (QDs)^14^, quantum Hall systems (‘chiral demon’)^15^, and even in a photonic setup^16^. Experimentally, Pekola *et al*.^17^ have reported creating Maxwell’s demon in a system of two quantum dots.

**Figure 1:**
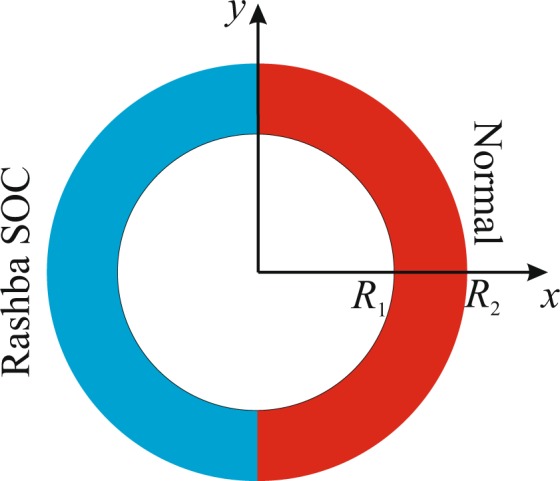
A non-reciprocal quantum ring where one arm contains the Rashba spin-orbit coupling (SOC) while that coupling is absent in the other arm.

Our nanoscale system, a *non-reciprocal* quantum ring (Fig. 1)^18,19^, where one arm of the ring contains the Rashba spin-orbit coupling (RSOC)^20–23^ while the other arm is normal, i.e., without the RSOC, is shown below to behave as if the demon is at work in pursuit of its assignment to break “one of the most perfect laws in physics”^2^. In what follows, we demonstrate that electrons can be channeled through the two arms according to their spin states by switching on and off (or reverse) an external magnetic field. Most significantly, this leads to different kinetic energies and effective spin temperatures in the two arms in the ground state, not unlike the expected signature of a Maxwell Demon. The demon neither infuses energy into the system or create entropy on its own, nor does it process information. In this sense it is quite different from the usual proposals^14–17^ based on a variant of Landauer’s principle^24^. Nevertheless it acts effectively as a one-way trapdoor between two spatially separated regions of the system.

## Results

Let us consider a non-reciprocal two-dimensional quantum ring (QR) with inner radius *R*_1_ and outer radius *R*_2_ and with the Rashba spin-orbit interaction^20–23^ that is applied only in one half of the ring. For simplicity we choose the confinement potential of the QR with infinitely high borders: *V*_conf_(*r*) = 0, if *R*_1_ ≤ *r* ≤ *R*_2_ and infinity otherwise.

### Model

The single-particle Hamiltonian of the system in an external magnetic field applied in the growth direction (the *z* direction, perpendicular to the ring) is

1 $${\mathscr{H}}=\frac{1}{2{m}_{e}}{{\boldsymbol{\Pi }}}^{2}+{V}_{{\rm{c}}{\rm{o}}{\rm{n}}{\rm{f}}}(r)+\frac{1}{2}g{\mu }_{{\rm{B}}}B{\sigma }_{z}+{{\mathscr{H}}}_{1},$$

where ${\boldsymbol{\Pi }}={\bf{p}}+\frac{e}{c}{\bf{A}}$, **A** = *B*/2(−*y*, *x*, 0) is the vector potential of the applied magnetic field along the *z* axis in the symmetric gauge. The third term on the right hand side of (1) is the Zeeman splitting. The last term describes the non-reciprocal RSOC

2 $${ {\mathcal H} }_{1}=f(\theta ){H}_{{\rm{SO}}}\,f(\theta )$$

where *f*(*θ*) = 1 if *π*/2 ≤ *θ* ≤ 3*π*/2 and 0 otherwise, is a function which describes the region of the QR where the RSOC is applied. *H*_SO_ is the RSOC Hamiltonian^20–23,25^

3 $${H}_{{\rm{SO}}}=\frac{\alpha }{\hslash }{[{\boldsymbol{\sigma }}\times {\boldsymbol{\Pi }}]}_{z}=i\frac{\alpha }{\hslash }(\begin{array}{ll}0 & {{\rm{\Pi }}}_{-}\\ -{{\rm{\Pi }}}_{+} & 0\end{array}),$$

with Π_±_ = Π_*x*_ ± *i*Π_*y*_ and *α* being the RSOC parameter which depends on the asymmetry in the *z* direction, generated either by the confinement or the electric field. The SOC coupling strength *α* can be experimentally determined for the QR materials^20–22^. Practical realization of the Rashba arm of the ring could perhaps be possible by appropriate covering of the ring by the gate electrode^26^.

We employ the exact diagonalization scheme^27^ in order to find the eigenvalues and eigenfunctions of the Hamiltonian (1). We take as basis states the eigenfunctions of the Hamiltonian (1) at *B* = 0 and *α* = 0^13,28,29^. The eigenfunctions of this Hamiltonian then have the form

4 $${\varphi }_{nl}(r,\theta )=\frac{C}{\sqrt{2\pi }}{e}^{{\rm{i}}l\theta }({J}_{l}({\gamma }_{nl}r)-\frac{{J}_{l}({\gamma }_{nl}{R}_{1})}{{Y}_{l}({\gamma }_{nl}{R}_{1})}{Y}_{l}({\gamma }_{nl}r))$$

where *n* = 1, 2, …, *l* = 0, ±1, ±2, … are quantum numbers, *J*_*l*_(*r*) and *Y*_*l*_(*r*) are Bessel functions of the first and second kind respectively, ${\gamma }_{nl}=\sqrt{2{m}_{e}{E}_{nl}/{\hslash }^{2}}$, where *E*_*nl*_ are the eigenstates defined from the boundary condition at *r* = *R*_2_, and the constant *C* is determined from the normalization integral.

In order to evaluate the energy spectrum and the wave functions of the Hamiltonian (1) we need to digonalize the matrix of the Hamiltonian (1) in a basis (4). Numerical calculations are performed for InAs QR with parameters^25^ *m*_*e*_ = 0.042*m*_0_ (*m*_0_ is the free electron mass), *g* = −14, *R*_1_ = 300 Å and *R*_2_ = 500 Å. In our calculations we have used 210 single-electron basis states |*n*, *l*, *s*〉 where *s* = ±1/2 is the electron spin, which is adequate for determining the first few energy eigenvalues of our system with high accuracy.

### Numerical results

**Figure 2:**
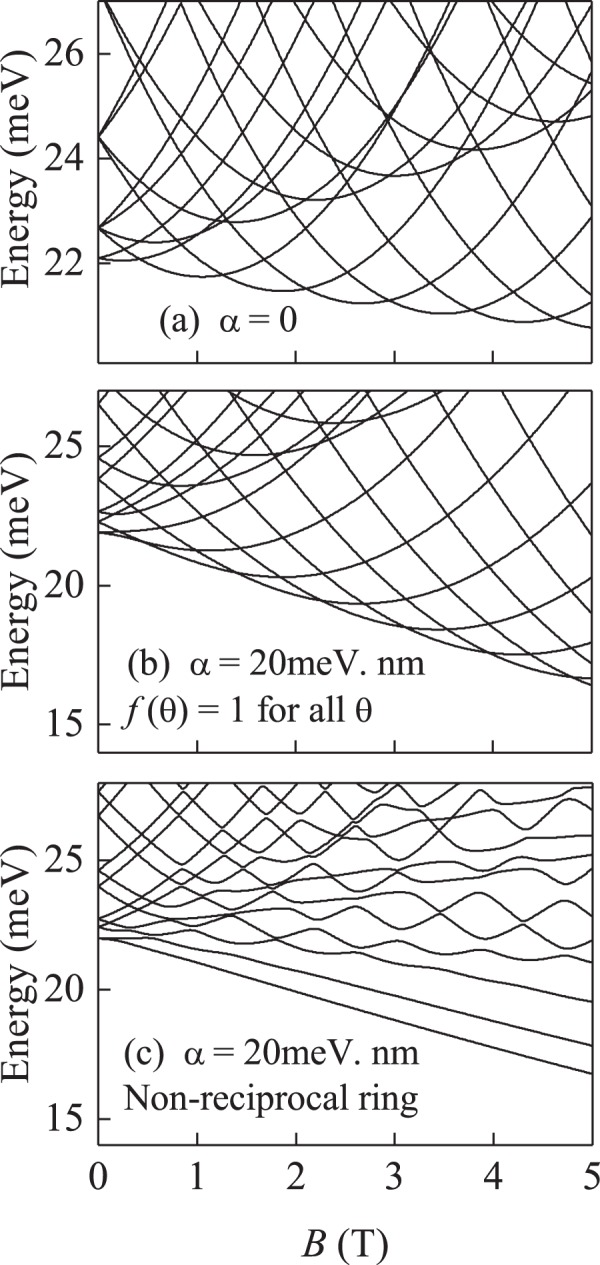
Magnetic field dependence of the electron energy spectra (**a**) for the QR without the RSOC, (**b**) for normal QR with the RSOC parameter *α* = 20 meV.nm and (**c**) for non-reciprocal QR with RSOC parameter *α* = 20 meV.nm.

In Fig. 2 the energy spectra of the system against the magnetic field is presented for various parameter values. For a QR without the RSOC [*α* = 0, Fig. 2(a)] the usual Aharonov-Bohm (AB) oscillations^13^ can be observed. With an increase of the magnetic field, the ground state periodically changes, as expected. For the normal QR with the RSOC [*f*(*θ*) = 1 for all *θ*, see Fig. 2(b)] the AB oscillations are still present, albeit with smaller amplitude but with the same period. The degeneracy of the energy levels at *B* = 0 being partially lifted, while the appearing level crossings as function of *B* are due to rotational invariance. This invariance is broken in the non-reciprocal ring and all degeneracies are lifted, leading to the complete disappearance of the AB oscillations in the ground state [Fig. 2(c)]. Clearly this means that the electron confinement inside the non-reciprocal ring is very different from that of the other two cases.

**Figure 3:**
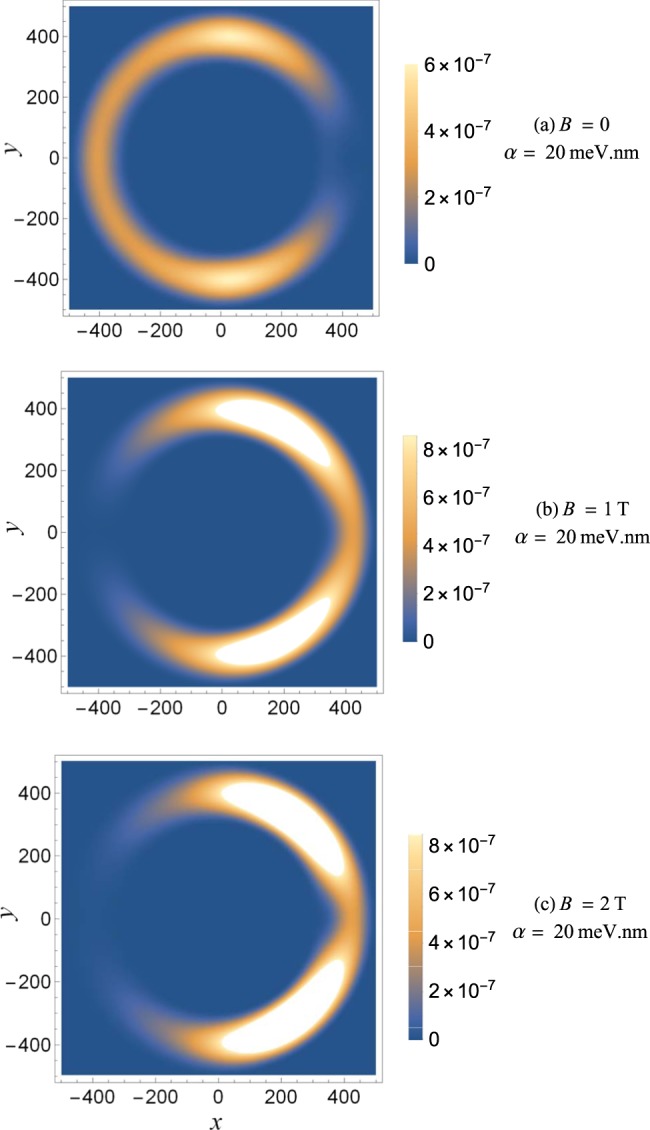
Ground state electron density in a QR with non-reciprocal RSOC for various values of the magnetic field.

In Fig. 3 the ground state electron density in a QR with non-reciprocal RSOC is presented for various values of the magnetic field. In the absence of the magnetic field [Fig. 3(a)] the electron charge is distributed all over the ring with a slight shift into the arm of the ring where the RSOC is present. However, when the magnetic field is increased, the electron moves to the areas of the ring where the RSOC is absent [Fig. 3(b,c)]. That is why we cannot observe the ground state AB oscillations in Fig. 2(c). The reason for this intriguing phenomena is that in a non-reciprocal QR the arm of the ring with RSOC is attractive for the spin-down states and repulsive for the spin-up states, as deduced from the average spin of the ground state 〈*S*_*z*_〉. Without the magnetic field the average spin of the ground state is almost zero because the RSOC mixes the spin-up and spin-down states. With increase of the magnetic field the average spin increases and is positive due to the Zeeman effect. Therefore the electron is confined in the non-Rashba arm of the ring.

**Figure 4:**
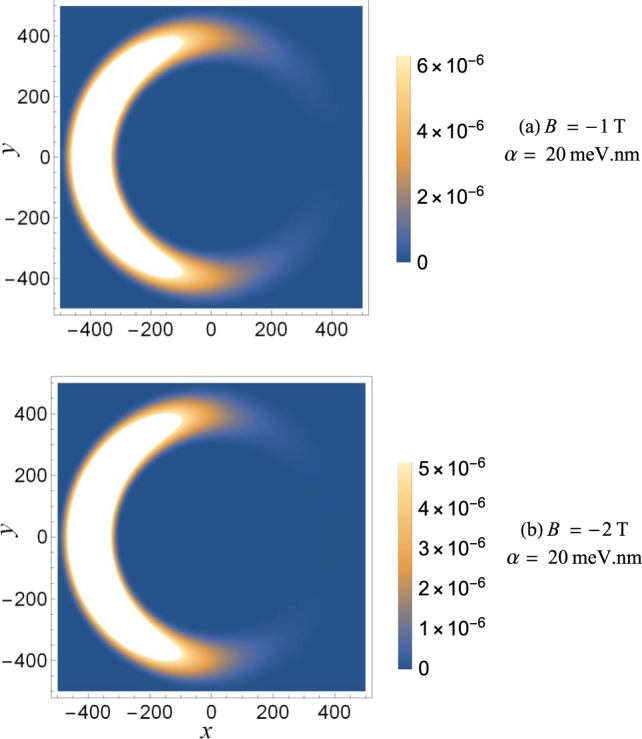
Same as in Fig. 3, but for opposite direction of the magnetic field.

Figure 4 is the same as Fig. 3 but for opposite direction of the magnetic field (negative values of *B*). In this case, when the magnetic field is increased, the average spin of the electron ground state is negative and now the electron is confined in the Rashba arm of the ring. Clearly, the magnetic field acts as the ‘trap door’ for the demon to sort the electrons according to their spins. The magnetic field can be used to control the electron spin of the ground state and thus the confinement of the electron in the ring.

### Spin temperatures and the “Maxwell’s demon”

**Figure 5:**
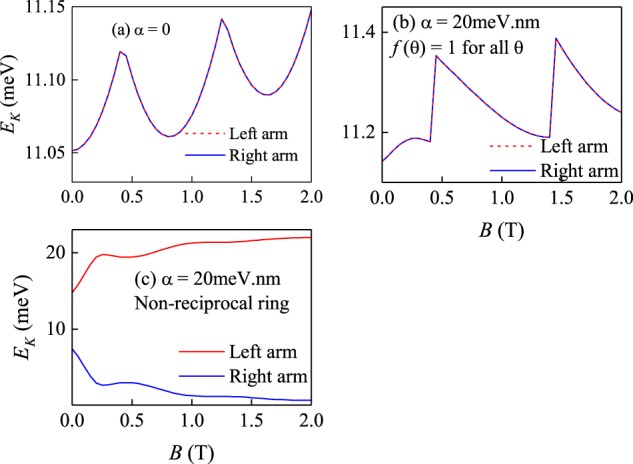
Expectation values of the kinetic energy operator in the two arms of the (**a**) Rashba free, (**b**) Rashba and (**c**) non-reciprocal ring as a function of the magnetic field.

Let us recall that Maxwell inferred the temperature difference in the two chambers of his gedankenexperiment from different expectation values of the kinetic energy (fast molecules in one chamber and slow molecules in the other chamber) using the standard Maxwell-Boltzmann distribution of a classical ideal gas. We shall now evaluate the corresponding quantity in our quantum system. The average kinetic energies in our system for the left and right arms of the ring can be defined as expectation values of the kinetic energy operator

5 $${E}_{K}^{L}={\int }_{{R}_{1}}^{{R}_{2}}\,{\int }_{\pi /2}^{3\pi /2}{\psi }^{\ast }(r,\theta )\frac{{{\rm{\Pi }}}^{2}}{2{m}_{e}}\psi (r,\theta )rdrd\theta $$

6 $${E}_{K}^{R}={\int }_{{R}_{1}}^{{R}_{2}}\,{\int }_{-\pi /2}^{\pi /2}{\psi }^{\ast }(r,\theta )\frac{{{\rm{\Pi }}}^{2}}{2{m}_{e}}\psi (r,\theta )rdrd\theta $$

where *ψ*(*r*, *θ*) is the ground state wave function of the system. The obtained average kinetic energies are shown in Fig. 5. The kinetic energies in the two arms of the normal and the Rashba ring are equal, but differ greatly for all *B* in case of the non-reciprocal ring. The reason for this behavior (not foreseeable by Maxwell) is the presence of a quantum degree of freedom (spin) which is used as a marker by the Hamiltonian itself (the “demon” is played by the magnetic field) to separate the particles with high and low kinetic energies into spatially distinct regions. It is therefore the simplest manifestation of a demon-like process without the interference of an intelligent being.The magnetic field could be turned on or reversed adiabatically, so that no entropy is generated during this process.

**Figure 6:**
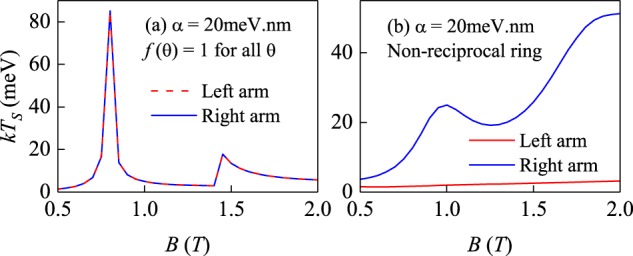
Spin temperatures in the two arms of the (**a**) Rashba and (**b**) non-reciprocal ring versus the magnetic field.

Similarly, we can also evaluate the average spin polarizations $\langle {S}_{z}^{L}\rangle$ and $\langle {S}_{z}^{R}\rangle$ for the left and right arms of the ring as expectation values of the spin operator for the ground state. To assess the action of our demon on the spin degree of freedom, we can define “spin temperature” *T*_*S*_, using only the Zeeman term in the spin Hamiltonian

7 $$\langle {S}_{z}\rangle =\frac{1}{2}\,\tanh (\frac{{{\rm{\Delta }}}_{z}}{k{T}_{S}}).$$

where Δ_*z*_ = *gμ*_*B*_*B*/2, and *k* is the Boltzmann constant. Equation (7) is used here to define a canonical “temperature” associated only with the spin degree of freedom for the arms of the ring, using the ground state values of the spin polarization. This yields two different spin temperatures in the two arms of the non-reciprocal ring (Fig. 6). Although these temperatures cannot be associated with the total system, which remains always at *T* = 0, the imbalance between the right and left arm can be expressed as different temperatures of a “spin gas” in the spirit of Maxwell^1^.

Once the spin temperatures of the different arms are obtained we can evaluate the “spin entropy” of the two arms^30^

8 $${S}_{spin}=k\{\mathrm{ln}[2\,\cosh (\frac{{{\rm{\Delta }}}_{z}}{k{T}_{S}})]-\frac{{{\rm{\Delta }}}_{z}}{k{T}_{S}}\,\tanh (\frac{{{\rm{\Delta }}}_{z}}{k{T}_{S}})\}.$$

**Figure 7:**
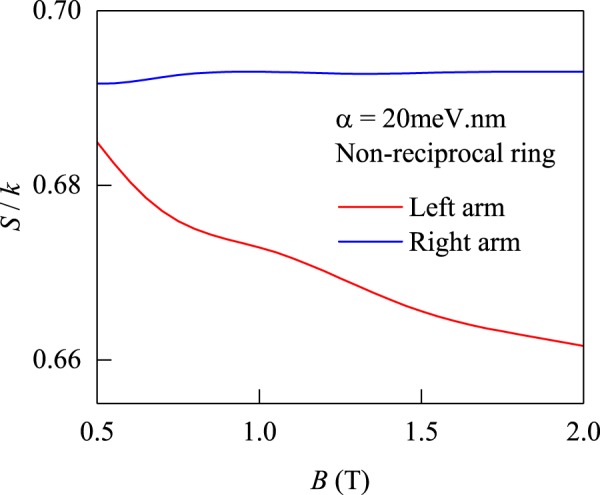
Spin entropy in the two arms of non-reciprocal ring versus the magnetic field.

It characterizes the demon’s action on the internal spin of the system. In Fig. 7, we notice a sharp drop in entropy of the “cool” arm (with the SOC) and a flat curve in the “hot” arm (without the SOC). Both temperature and entropy of the spin subsystem indicate the sorting action of the ring but not a deviation from equilibrium of the total system. It is well-known that the density matrix of a subsystem *A* is usually mixed (*S*_*A*_ > 0) while the total system remains in a pure state *S*_*tot*_ = 0. In our case, the spin degree of freedom acquires a non-zero entropy because it is entangled with the spatial degrees of freedom through the Rashba-coupling. The crucial difference between the inhomogeneous Rashba-ring and an inhomogeneous potential (which would also lead to a spatially varying electron density) is the fact that the potential *V*_conf_(*r*) is constant along the angle *θ*. The spin, acting as a quantum marker, is “measured” by the magnetic field without recording of information and causes the sorting in both halves of the ring.

## Conclusions and Discussions

In a non-reciprocal QR, the AB oscillations disappear for all values of the magnetic field. The electron density indicates that, for zero magnetic field the electron is mostly located in the Rashba arm of the ring. However, in a non-zero magnetic field the electron moves to the non-Rashba arm of the ring. The Rashba arm is attractive for states with negative average spin polarization and repulsive for states where the average spin polarization is positive. Of course all of our states are mixtures of spin-up and spin-down states due to the SOC. The ground state electron density indicates that, with an increase of the magnetic field the value of the average spin for the ground state is positive and increases. Hence the electron is confined in the non-Rashba arm of the ring. For negative values of the magnetic field the situation is reversed.

The non-reciprocal QR is clearly a manifestation of demon’s workplace: the electron sorting in different arms of the ring according to their spins leads to different kinetic energies and spin temperatures in the two arms of the ring. This unusual sorting by the demon takes place through the spin, which is a pure quantum character and does not have any classical counterpart. Observation of the absence of AB oscillations in a non-reciprocal QR for all values of the magnetic field, and the different temperatures in the two arms would strongly indicate the *action*, if not necessarily the *presence* of the “restless and lovable poltergeist”^31^ in our nanoscale system. The Coulomb interaction might play an important role in our ring containing multiple electrons, although AB oscillations are a single-particle effect.

In the experiment involving two quantum dots^17^ mentioned above, the system-demon interaction was mediated by the Coulomb potential. Our present approach is well suited to study also the case of interacting electrons very accurately in a non-reciprocal QR. We have confined our analysis to ground state properties. Obviously, a generalization to finite temperatures and especially to real-time dynamics is necessary to investigate possible consequences for the second law of thermodynamics, which has not been impacted by our results. However, the demon-like sorting action appears already at *T* = 0 in our present system and might turn out to be useful in algorithmic cooling^32–34^ and ‘spin refrigeration’^35^ in quantum information science.

Although we have considered InAs quantum ring as our model system, for experimental realization of non-reciprocal quantum rings the material of choice could also be graphene^36–43^. Graphene quantum rings have been studied theoretically^44,45^ and experimentally^46^, where observation of AB oscillations was reported. The Rashba SOC in graphene is negligibly small^47^. However, there are several proposals in the literature about enhancement of Rashba SOC in graphene^48,49^, that could perhaps help to create the Rashba arm of the ring.

## Methods

We use the basis in Eq. (4) to apply the exact diagonalization scheme for the Hamiltonian (1). We use 210 single-electron basis states and numerically evaluate all the corresponding matrix elements of the Hamiltonian (1). The eigenvalues and eigenfunctions of the 210 × 210 matrix have been evaluated using the standard numerical diagonalization algorithms^27–29^. The exact diagonalization scheme has been widely used by many other authors. In this method the energy eigenvalues of complex quantum structures are evaluated with desired accuracy by appropriately increasing the number of basis states. This method can therefore be considered as exact (numerically). In our present work, the number of basis states chosen is sufficient to evaluate the eigenvalues with very high accuracy. The ground state wave functions, the kinetic energies, the spin temperature, and the spin entropy are obtained by numerically evaluating the integrals.
